# Non-steroidal anti-inflammatory drug-induced apoptosis in gastric cancer cells is blocked by protein kinase C activation through inhibition of c-*myc*

**DOI:** 10.1038/sj.bjc.6690062

**Published:** 1999-02

**Authors:** G H Zhu, B C Y Wong, M C Eggo, C K Ching, S T Yuen, E Y T Chan, K C Lai, S K Lam

**Affiliations:** Department of Medicine, Queen Mary Hospital, University of Hong Kong, Hong Kong; Department of Medicine, the University of Birmingham, Birmingham, UK; Department of Pathology, Queen Mary Hospital, University of Hong Kong, Hong Kong

**Keywords:** non-steroidal anti-inflammatory drugs, gastric cancer, apoptosis, antiproliferation, oncogene, protein kinase C, cell cycle

## Abstract

Apoptosis plays a major role in gastrointestinal epithelial cell turnover, ulcerogenesis and tumorigenesis. We have examined apoptosis induction by non-steroidal anti-inflammatory drugs (NSAIDs) in human gastric (AGS) cancer cells and the role of protein kinase C (PKC) and apoptosis-related oncogenes. After treatment with aspirin or indomethacin, cell growth was quantified by MTT assay, and apoptosis was determined by acridine orange staining, DNA fragmentation and flow cytometry. The mRNA and protein of p53, p21^waf1/cip1^ and c-*myc* was detected by Northern and Western blotting respectively. The influence of PKC on indomethacin-induced apoptosis was determined by co-incubation of 12-*O*-tetradecanoylphorbol 13-acetate (TPA). The role of c-*myc* was determined using its antisense oligonucleotides. The results showed that both aspirin and indomethacin inhibited cell growth and induced apoptosis of AGS cells in a dose- and time-dependent manner, without altering the cell cycle. Indomethacin increased c-*myc* mRNA and protein, whereas p53 and p21^waf1/cip1^ were unchanged. Down-regulation of c-*myc* by its antisense oligonucleotides reduced apoptosis induction by indomethacin. TPA could inhibit indomethacin-induced apoptosis and accumulate cells in G_2_/M. Overexpression of c-*myc* was inhibited by TPA and p21^waf1/cip1^ mRNA increased. In conclusion, NSAIDs induce apoptosis in gastric cancer cells which may be mediated by up-regulation of c-*myc* proto-oncogene. PKC activation can abrogate the effects of NSAIDs by decreasing c-*myc* expression. © 1999 Cancer Research Campaign


*British Journal of Cancer* (1999) **79**(3/4), 393–400
© 1999 Cancer Research Campaign
Article no. bjoc.1998.0062

### Non-steroidal anti-inflammatory drug-induced apoptosis in gastric cancer cells is blocked by protein kinase C activation through inhibition of c-*myc*

**GH Zhu[1], BCY Wong[1], MC Eggo[2], CK Ching[1], ST Yuen[3], EYT Chan[3], KC Lai[1] and SK Lam[1]**

[1]Department of Medicine, Queen Mary Hospital, University of Hong Kong, Hong Kong; [2]Department of Medicine, the University of Birmingham, Birmingham, UK; [3]Department of Pathology, Queen Mary Hospital, University of Hong Kong, Hong Kong

**Summary** Apoptosis plays a major role in gastrointestinal epithelial cell turnover, ulcerogenesis and tumorigenesis. We have examined apoptosis induction by non-steroidal anti-inflammatory drugs (NSAIDs) in human gastric (AGS) cancer cells and the role of protein kinase C (PKC) and apoptosis-related oncogenes. After treatment with aspirin or indomethacin, cell growth was quantified by MTT assay, and apoptosis was determined by acridine orange staining, DNA fragmentation and flow cytometry. The mRNA and protein of p53, p21$^{waf1/cip1}$ and c-*myc* was detected by Northern and Western blotting respectively. The influence of PKC on indomethacin-induced apoptosis was determined by co-incubation of 12-*O*-tetradecanoylphorbol 13-acetate (TPA). The role of c-*myc* was determined using its antisense oligonucleotides. The results showed that both aspirin and indomethacin inhibited cell growth and induced apoptosis of AGS cells in a dose- and time-dependent manner, without altering the cell cycle. Indomethacin increased c-*myc* mRNA and protein, whereas p53 and p21$^{waf1/cip1}$ were unchanged. Down-regulation of c-*myc* by its antisense oligonucleotides reduced apoptosis induction by indomethacin. TPA could inhibit indomethacin-induced apoptosis and accumulate cells in $G_2$/M. Overexpression of c-*myc* was inhibited by TPA and p21$^{waf1/cip1}$ mRNA increased. In conclusion, NSAIDs induce apoptosis in gastric cancer cells which may be mediated by up-regulation of c-*myc* proto-oncogene. PKC activation can abrogate the effects of NSAIDs by decreasing c-*myc* expression.

**Keywords:** non-steroidal anti-inflammatory drugs; gastric cancer; apoptosis; antiproliferation; oncogene; protein kinase C; cell cycle

Non-steroidal anti-inflammatory drugs (NSAIDs) are among the most commonly prescribed medications that can be obtained over the counter. Apart from their well-known analgesic, antipyretic and anti-inflammatory effects, they have also been used in the treatment of familial adenomatous polyposis (Giardiello et al, 1993). Sulindac, in particular, has been demonstrated in several epidemiological studies to significantly reduce the incidence of colonic adenomas and adenocarcinomas (Marnett, 1992; Giovannucci et al, 1994). Aspirin use was inversely associated with fatal cancers of the oesophagus and stomach as well as colon and rectum, but not generally with fatal cancers outside the digestive tract (Thun et al, 1993). Record linkage studies in Finland and Sweden found decreased risk of stomach and colorectal cancers, but not oesophagus in patients with rheumatoid arthritis taking high doses of aspirin and other NSAIDs (Isomaki et al, 1978; Gridley et al, 1993). NSAIDs, such as indomethacin, have been shown to inhibit the growth of colon cancer cell lines and induce apoptosis in vitro, which was considered to be an important mechanism in their antineoplastic activity (Hanif et al, 1996; Shiff et al, 1996). Furthermore, apoptosis has been implicated to play a vital role in gastric epithelial cell turnover, ulcerogenesis and even carcinogenesis (Hall et al, 1994; Spyridon et al, 1994). A study of the effects of NSAIDs on apoptosis of gastric epithelial cells and its underlying intracellular events could present novel points for therapeutic intervention in gastropathy.

*Received 9 January 1998*
*Revised 19 May 1998*
*Accepted 21 May 1998*

*Correspondence to:* BCY Wong, Division of Gastroenterology and Hepatology, Department of Medicine, University of Hong Kong, Queen Mary Hospital, Hong Kong

Protein kinase C (PKC), a serine–threonine kinase, is a family of 12 isoenzymes which mediate phosphorylation of numerous protein substrates and play a central role in signalling pathways regulating cell differentiation, tumour promotion and apoptosis (Clemens et al, 1992; Lucas and Sanchez-Margalet, 1995; Deacon et al, 1997). Phorbol esters, such as 12-*O*-tetradecanoylphorbol 13-acetate (TPA), activate PKC in vitro in a manner similar to the physiological activator diacylglycerol (DAG) and have, therefore, been widely used to study the function of PKC. Both induction and inhibition of apoptosis have been reported in different cell lines treated with TPA (Lucas and Sanchez-Margalet, 1995). These contradictory findings may be because it can activate several PKC isoenzymes. Unlike the physiological activator DAG, TPA is a stable compound which will induce a persistent activation of PKC leading to its down-regulation. The PKC isoenzymes are differentially sensitive to down-regulation. So, the diverse biological effects of phorbol esters are related to the differential activation or down-regulation of one or more of the isoenzymes (Deacon et al, 1997).

Oncogenes and tumour-suppressor genes are well documented to be involved in mediating apoptosis (Liebermann et al, 1995; Hale et al, 1996). p53 is a transcription factor which stimulates the synthesis of inhibitors of cyclin-dependent kinases (CDKs), such as p21$^{waf1/cip1}$ which binds to CDKs and inhibits their action, thereby, blocking cell proliferation (Xiong et al, 1993; El Deiry et al, 1994). When mammalian cells are exposed to ionizing irradiation or other DNA-damaging agents, the cells are arrested at $G_1$

phase until the genomic lesions are fully repaired. If the damage to DNA is irreparable, p53 triggers apoptosis. Cells without p53 or with mutant p53 genes lack this checkpoint and cells enter S-phase without appropriate DNA repair, leading to fixation and propagation of genetic alteration (Hale et al, 1996). The c-*myc* proto-oncogene plays a role in the control of cell proliferation, differentiation as well as apoptosis (Askew et al, 1991; Evans et al, 1992). Deregulated expression of c-*myc* can induce apoptosis in conjunction with growth-regulating signals, such as growth factor deprivation, high cell density and chemotherapeutic treatment (Evans et al, 1992; Lotem and Sachs, 1993). c-*myc* antisense oligodeoxynucleotides can inhibit the intracellular expression of the targeted gene specifically because of the exquisite specificity of the Watson–Crick base-pair interactions that result in the formation of mRNA–DNA hybrid structure. Antisense techniques have been widely used in studying the role of c-*myc* in apoptosis.

In this study, we have tested the effects of aspirin and indomethacin on a gastric (AGS) cancer cell line and investigated the roles of both drugs on cell growth and apoptosis. The mechanisms associated with these changes were also studied, including the PKC signalling pathways and apoptosis-related genes including p53, $p21^{waf1/cip1}$ and c-*myc*.

### MATERIALS AND METHODS

### Cell culture

AGS, a poorly differentiated gastric adenocarcinoma cell line, was purchased from American Type Culture Collection (ATCC, Rockville, MD, USA) and used in the present study. Cells were passaged into RPMI-1640 medium containing 10% fetal bovine serum (FBS), 100 U $ml^{-1}$ penicillin, 100 μg $ml^{-1}$ streptomycin (Gibco BRL, Life Technologies, NY, USA). Cells were cultured in 25 $cm^2$ culture flasks (Corning, New York, NY, USA) as a monolayer in a 95% air/5% carbon dioxide humidified atmosphere at 37°C.

Both indomethacin and aspirin (Sigma, St Louis, MO, USA) were freshly prepared in absolute ethanol before use. TPA (CalBiochem, La Jolla, CA, USA) was prepared in DMSO. Stock preparations of the reagents were stored at –20°C. Vehicle controls of absolute ethanol and DMSO (less than 0.1%) were included in the studies. Cells were incubated for 24 h with different concentrations of indomethacin, aspirin, and TPA alone or in combination. After treatment, adherent cells were removed by trypsinization and combined with the floating cells in the medium (Hanif et al, 1996). Cells were collected by centrifugation for further analysis. Antisense and sense oligonucleotides for c-*myc* were purchased from Biognostik (Germany). For oligonucleotide experiments, cells growing in log phase were seeded in 24-well plates at a density of $1 \times 10^5$ cells per well. Specified concentrations of oligonucleotides were added to the cell culture for 14 h, then indomethacin (400 μM) was added to the culture for a fixed time period for further analysis.

### MTT assay

Antiproliferation effects were measured by a modified MTT assay (Carmichael et al, 1988). The assay detects living but not dead cells, and the signal generated is dependent on the degree of activation of the cells. About 5000 cells per well were plated in 96-well microtitre plates and incubated overnight in 100 μl of culture media. Then cells were treated with various concentrations of indomethacin or aspirin for fixed time intervals. Ten microlitres of stock MTT (2.5 mg $ml^{-1}$) was then added to each well, and the cells were further incubated at 37°C for 4 h. The supernatant was removed and 100 μl of 0.04 M hydrochloric acid in isopropanol was added to each well. The absorbance at a wavelength of 595 nm was measured by a microELISA reader (BioRad, USA). The negative control well has no cells, but the culture medium only.

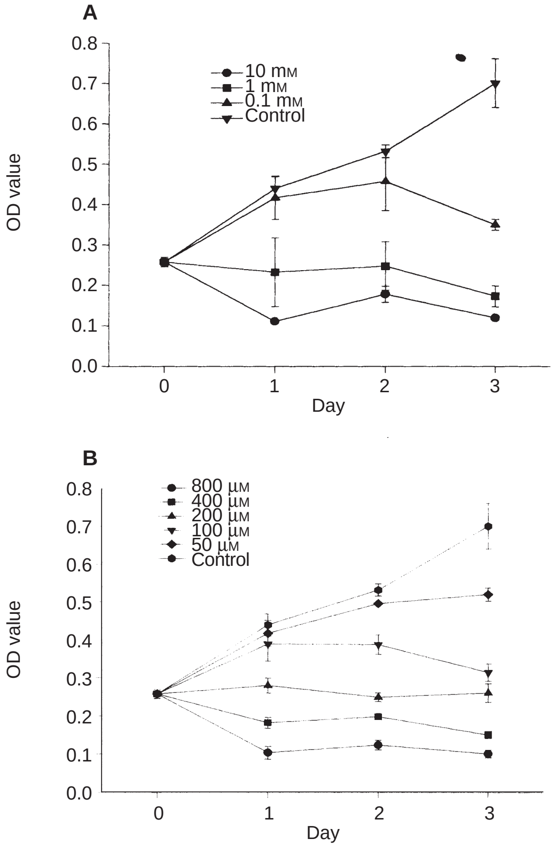

**Figure 1** Dose–response of aspirin and indomethacin on growth of AGS cells. Cells were treated with various concentration of aspirin (**A**) or indomethacin (**B**) for different time periods. The cell proliferation was determined by MTT assay. The values are expressed as means ± s.d. ($n > 3$)

### Flow cytometry

Cells from both treatment and control groups were collected and washed with ice-cold phosphate buffered saline (PBS, pH 7.4). They were fixed in ice-cold 70% ethanol in PBS and stored at –20°C. Before analysis, the cells were washed and resuspended in PBS. One hundred microlitres of RNAase I (1 mg $ml^{-1}$) and 100 μl of propidium iodide (PI, 400 μg $ml^{-1}$, Sigma) were added and incubated at 37°C for 30 min. The analysis of samples was performed by a flow cytometry (Coulter Epics XL, UK). The cell cycle phase distribution was calculated from the resultant DNA histogram using Multicycle AV software (Phoenix Flow System, San Diego, CA, USA) and expressed as a percentage of cells in $G_0/G_1$, S- and $G_2/M$ phases. The apoptotic cells can be observed on a DNA histogram as a subdiploid or 'pre-$G_1$' peak (Darzynkiewicz et al, 1992).

 © Cancer Research Campaign 1999

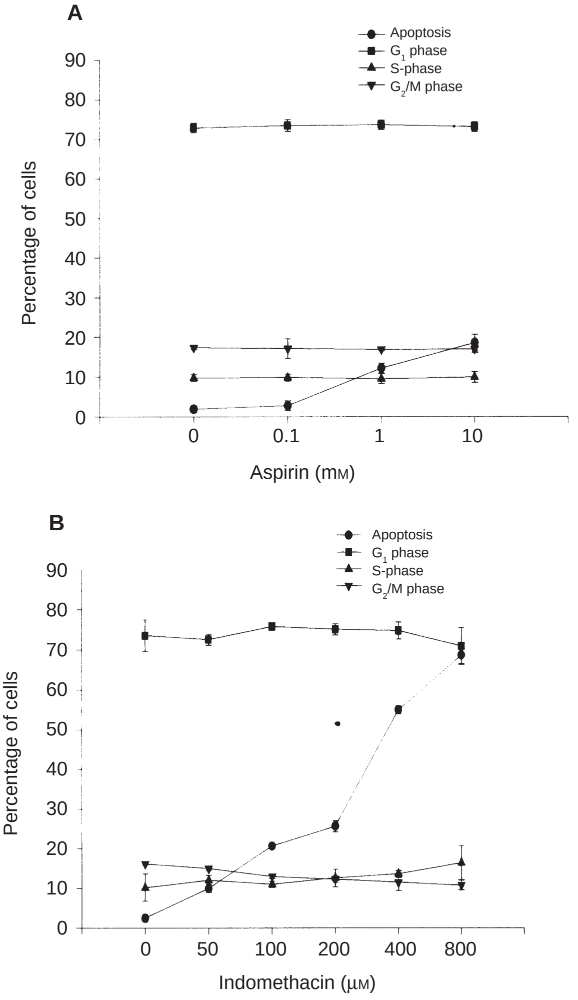

**Figure 2** Cell cycle phase distribution and apoptosis of AGS cells treated with aspirin and indomethacin. Cells were treated with various concentrations of aspirin (**A**) or indomethacin (**B**) for 24 h and harvested. The cell cycle phase distribution and apoptosis was determined by FACS analysis. The values are expressed as means ± s.d. ($n > 3$)

### DNA fragmentation analysis

DNA fragmentation was analysed as described previously (Grant et al, 1992) with some modification. Briefly, after treatment with various agents as described above, cells were harvested and washed twice in ice-cold PBS. The final pellet was lysed in 10 mM tris-HCl (pH 7.4) buffer containing 25 mM EDTA, 0.5% SDS and 0.1 mg ml$^{-1}$ proteinase K (Sigma) and incubated at 50°C for 12–18 h. DNA was extracted with an equal volume of phenol/chloroform/isoamyl alcohol (25:24:1) and precipitated with two volumes of ice-cold absolute ethanol and 1/10 volume 3 M sodium acetate. DNA was collected, washed once with 70% ethanol, and dissolved in TE buffer (10 mM Tris and 1 mM EDTA, pH 8.0). DNA samples were incubated with 10 mg ml$^{-1}$ RNAase I (Sigma) for 1 h at 37°C. Equal amounts (10 μg per well) of DNA was electrophoresed in 1.8% agarose gels impregnated with ethidium bromide (0.1 μg ml$^{-1}$) for 2 h at 60 V. DNA marker (50–1000 bp, FMC, Rockland, ME, USA) was run at the same time. DNA fragments in the form of laddering pattern were visualized by ultraviolet transillumination.

### Acridine orange staining

Single-cell suspensions were fixed in 1% formalin/PBS and stained with acridine orange (AO, 10 μg ml$^{-1}$, Sigma). A drop of the stained cell suspension was placed on a microscope slide. Cells were visualized under UV fluorescence microscope with blue–green filter, as described previously (Elder et al, 1996). Apoptotic cells were defined as cells showing cytoplasmic and nuclear shrinkage and chromatin condensation or fragmentation morphologically. At least 300 cells were counted and the percentage of apoptotic cells was determined.

### RNA preparation and Northern blot analysis

Total cellular RNA was isolated following the methods as described previously (Rhoads, 1975) after some modification. Briefly, cells were washed with ice-cold PBS, lysed with 4 M lithium chloride/8 M urea/6 mM EDTA and then precipitated overnight at 4°C. After centrifugation for 15 min at 13 000 r.p.m. at 4°C, the RNA pellet was dissolved in DEPC-treated water, extracted with phenol/chloroform/isoamyl alcohol (25:24:1), and then precipitated with ethanol and sodium acetate. Northern blot analysis was performed according to the method previously described (Ausubel et al, 1995). Equal amounts (20 μg) of total RNA per lane were electrophoresed on 1.0% denaturing formaldehyde agarose gel and transferred onto nylon membranes (Amersham, UK). The cDNA probes of *p53*, c-*myc* (Oncogene Research Products, Cambridge, MA, USA) and waf1 probe from pSXV-WAF1 (ATCC) excised by *Eco*RI and *Xho*I (New England Biolabs, Beverly, MA, USA) were radiolabelled with [α-$^{32}$P]dCTP (Amersham) by the Megaprime random prime labelling system (Amersham). After hybridization with a different probe overnight at 42°C, the membrane was washed with 2.0 × sodium chloride, sodium phosphate EDTA buffer (SSPE)/0.1% SDS, 1.0 × SSPE/0.1% SDS and 0.2 × SSPE/0.1% SDS at 42°C, and exposed to radiographic film (Kodak, Rochester, NY, USA) at –70°C. The membrane was reprobed for use in another hybridization in which a glyceraldehyde-3-phosphate dehydrogenase (GAPDH) mRNA served as an internal control. GAPDH oligonucleotide probe (Oncogene Research Products) was radiolabelled with [γ-$^{32}$P] ATP by 5′-end labelling kit (Amersham).

### Western blot analysis

After the drug treatment, the cells were extracted with lysis buffer containing 1% Triton × 100, 50 mM sodium chloride, 50 mM sodium fluoride, 20 mM Tris, pH 7.4, 1 mM EDTA, 1 mM EGTA, 1 mM sodium vanadate, 0.2 mM PMSF and 0.5% NP-40. The protein concentration was determined by using Bicinchoninic acid assay (BCA) (Smith et al, 1985) with bovine serum albumin (Sigma) as standard. Western blotting was carried out as described previously (Li et al, 1996). In brief, equal amount of cell lysates (60 μg) were solubilized in sample buffer by boiling for 5 min and subjected to 10% sodium dodecyl sulphate polyacrylamide gel electrophoresis (SDS-PAGE) followed by electrotransfer onto a nitrocellulose membrane (Sigma). The membrane was incubated first with an appropriate primary antibody and then with peroxidase-conjugated anti-mouse IgG antibody in the second reaction. The membranes were washed and developed by chemiluminescence (ECL) Western blot detection system (Transduction Laboratories, Lexington, KY, USA) and exposed to film. Mouse monoclonal antibodies p53 (Ab-6), p21$^{waf1/cip1}$ (Ab-1), c-*Myc* (Ab-1) and peroxidase-conjugated anti-mouse IgG were purchased from Oncogene Research Products.

© Cancer Research Campaign 1999

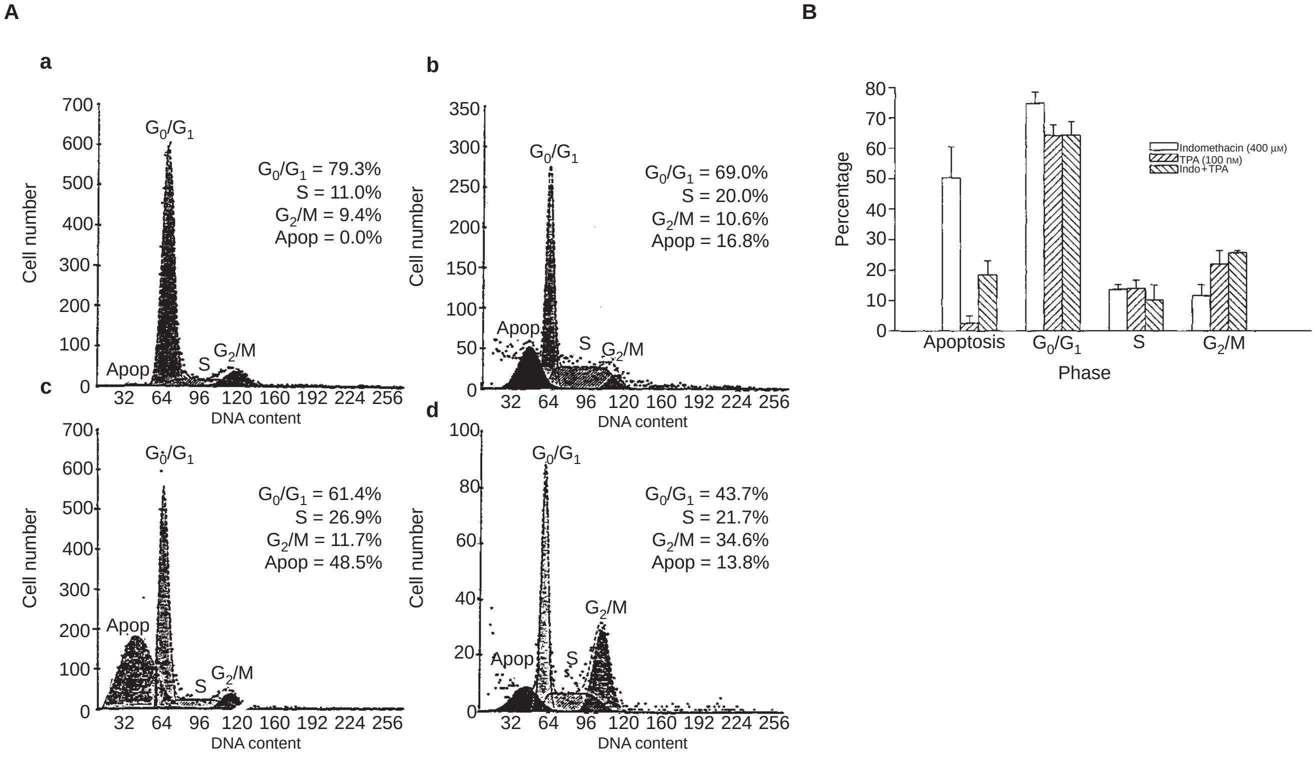

**Figure 3** The results of FACS analysis of AGS cells treated with aspirin and indomethacin with or without TPA. Cells were treated with aspirin, indomethacin and TPA alone or in combination for 24 h and their DNA content was determined by FACS, as described in Materials and methods. (**A**) DNA histogram. (**a**) control; (**b**) 1 mM aspirin; (**c**) 400 µM indomethacin; (**d**) 400 µM indomethacin + 100 nM TPA; (**B**) TPA on apoptosis and cell kinetics of indomethacin-treated AGS cells. The values are expressed as means ± s.d. ($n > 3$)

### Statistical analysis

The data shown were mean values of at least three different experiments and expressed as means ± s.d. Student's *t*-test was used to compare the results. A *P*-value of less than 0.05 is considered statistically significant.

### RESULTS

### Effects of aspirin and indomethacin on cell growth

To evaluate the effects of aspirin and indomethacin on the growth of gastric cancer cells in vitro, cells were seeded in 96-well culture plates at a density of 5000 cells per well. Various concentrations of aspirin (0.1–10 mM) and indomethacin (50–800 µM) were added in the culture medium for 1, 2 or 3 days. Cell growth was determined by MTT assay. As Figure 1 shows, both indomethacin and aspirin could inhibit gastric cancer cell growth in a dose- and time-dependent manner. Indomethacin showed a more potent effect on reducing AGS cell growth compared with aspirin.

### Effect of aspirin and indomethacin on the cell cycle phase distribution

To investigate the antiproliferation mechanisms of aspirin and indomethacin, we studied the effect of these compounds on cell cycle phase distribution of gastric adenocarcinoma cells using FACS analysis. The results showed that after the indicated treatments for 24 h, neither aspirin nor indomethacin interfered with cell cycle phase distribution of AGS cells when compared with the control (Figure 2).

### Apoptosis of gastric cancer cells induced by aspirin and indomethacin

Apoptosis of gastric cancer cells after treatment was evaluated by three different methods as described above: (1) measurement of DNA content of cells by PI staining and FACS analysis to detect the pre-$G_1$ peak; (2) agarose gel electrophoresis of genomic DNA to detect DNA fragmentation; (3) AO staining to detect the typical morphological changes under fluorescence microscopy. The FACS analysis showed that both aspirin and indomethacin could induce apoptosis in AGS cells, with a typical subdiploid peak on the histogram (Figure 3A). Apoptosis induction followed a dose-dependent and non-linear manner (Figure 2). Compared with indomethacin, aspirin was less potent in inducing apoptosis of AGS cells. It failed to induce apoptosis at ≤0.1 mM. DNA fragmentation in AGS cells treated with aspirin (1 mM, 10 mM) and indomethacin (100–800 µM) was shown as a ladder pattern on agarose gel (Figure 4). The AO staining showed that both aspirin and indomethacin could induce apoptosis in AGS cells morphologically, which was characterized by cytoplasmic and nuclear shrinkage, chromatin condensation and fragmentation (Figure 5).

### Effect of 12-*O*-betradecanoyl phorbol 13-acetate (TPA) on indomethacin-induced apoptosis and cell cycle

To study whether the PKC pathway is mediated in apoptosis induction by indomethacin, we investigated the effect of the PKC activator TPA, a pan-activator of PKC isoenzymes affecting all but the atypical class of PKCs (ζ, λ, µ) (Jarvis et al, 1994), on

 © Cancer Research Campaign 1999

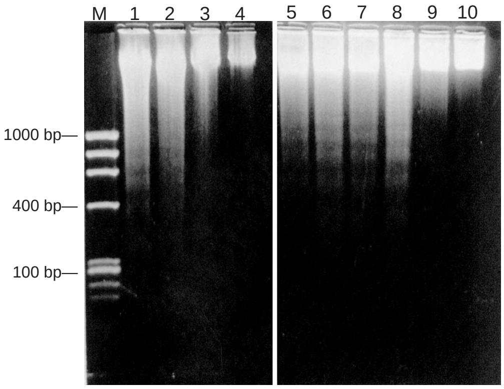

**Figure 4** Induction of DNA fragmentation by indomethacin and aspirin. AGS cells were exposed to the agents indicated for 24 h and the formation of oligonucleosomal fragments was determined by agarose gel electrophoresis as described in the text. M, DNA marker; lanes 1–3, aspirin 10, 1, 0.1 mM; lane 4, control. Lanes 5–9, indomethacin 800, 400, 200, 100, 50 μM; lane 10, control indomethacin-induced apoptosis of AGS cells. As a result, the proportion of cells in the $G_0/G_1$ phase was significantly decreased and that in $G_2$/M phase was increased after chronic treatment with TPA alone at 100 nM for 24 h (Figure 3B). Indomethacin-induced apoptosis was inhibited by co-incubation with 100 nM TPA, decreasing from 50.2±10% to 16.4±6.7%. In addition, cells at $G_0/G_1$ phase were reduced from 74.8±3.6% to 60.6±4.7% ($P$<0.05) and those at $G_2$/M phase was increased from 11.5±3.6% to 33.6±6.6% ($P$<0.01) (Figure 3B).

### Effects of indomethacin on p53, p21$^{waf1/cip1}$ and c-*myc* mRNA level

The mRNA level of p53, p21$^{waf1/cip1}$ and c-*myc* was determined by Northern blotting in indomethacin-treated AGS cells. Cells exposed to 400 μM of indomethacin resulted in a progressive increase in c-*myc* mRNA levels noted as early as 2 h and reached a maximum at 8 h, whereas p53 and p21$^{waf1/cip1}$ mRNA remained unchanged over time. Equal amounts of RNA were loaded in each lane, as determined by hybridization with the internal control GAPDH probe (Figure 6A).

### Effects of indomethacin on p53, p21$^{waf1/cip1}$ and c-*myc* protein level

The protein levels of p53, p21$^{waf1/cip1}$ and c-*myc* were determined by Western blotting in indomethacin-treated AGS cells. Cells treated with 400 μM of indomethacin resulted in a marked increase in c-*myc* protein level noted as early as 4 h and reached a maximum at 8 h, whereas p53 and p21$^{waf1/cip1}$ protein levels remained unchanged (Figure 6B).

### Effect of TPA on p53, p21$^{waf1/cip1}$ and c-*myc* mRNA level in indomethacin-treated AGS cells

Figure 7 shows that 100 nM TPA greatly inhibited the overexpression of c-*myc* mRNA induced by indomethacin (400 μM) at 8 h. However, p21$^{waf1/cip1}$ mRNA was increased at 8 h, whereas p53 mRNA remained unchanged. Equal amounts of RNA were loaded in each lane, as determined by hybridization with the internal control GAPDH probe.

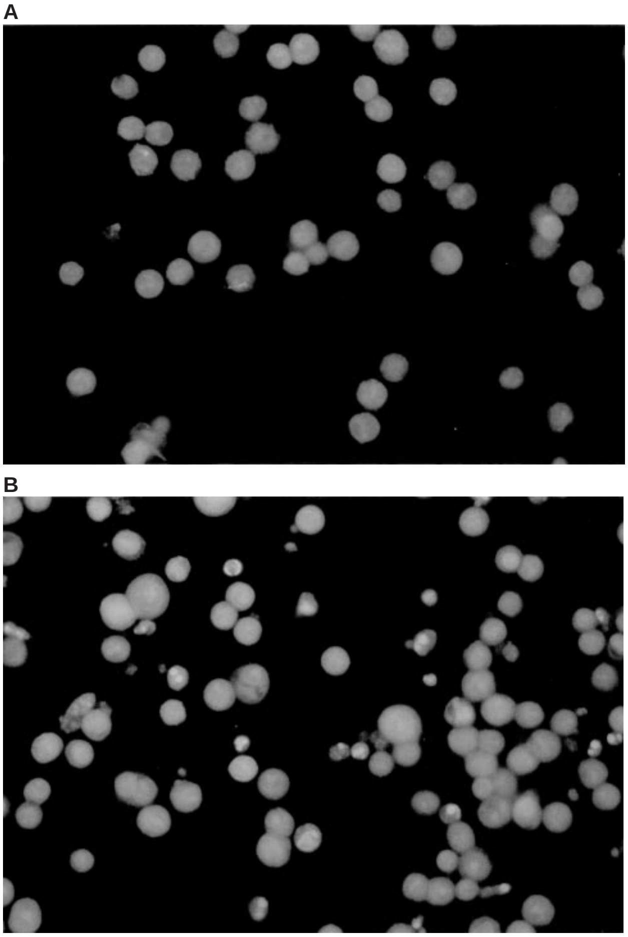

**Figure 5** Fluorescence photograph of AGS cells treated with indomethacin. Cells were treated with 400 μM indomethacin for 24 h. The cells were stained with acridine orange and examined under fluorescence microscopy. (**A**) control; (**B**) treated with indomethacin

### Down-regulation of c-*myc* with its antisense oligonucleotides on indomethacin-induced apoptosis

The role of c-*myc* proto-oncogene in indomethacin-induced apoptosis was confirmed using various concentrations of antisense oligonucleotides. Figure 8 shows that 2 μM c-*myc* antisense oligonucleotide could inhibit the indomethacin-induced increase in c-*myc* protein at 8 h, whereas sense oligonucleotide, as a random control, showed no effect on the c-*myc* overexpression. c-*myc* antisense oligonucleotide could inhibit the indomethacin-induced apoptosis. At 400 μM of indomethacin, the percentage of apoptotic cells decreased from 50.3±3.2% to 24.7±2.1% by the addition of 2 μM of antisense oligonucleotide. The same concentration of c-*myc* sense oligonucleotides showed no effect on apoptosis induced by indomethacin.

### DISCUSSION

It has been demonstrated for the first time in this study that both aspirin and indomethacin can inhibit the growth of AGS gastric

© Cancer Research Campaign 1999

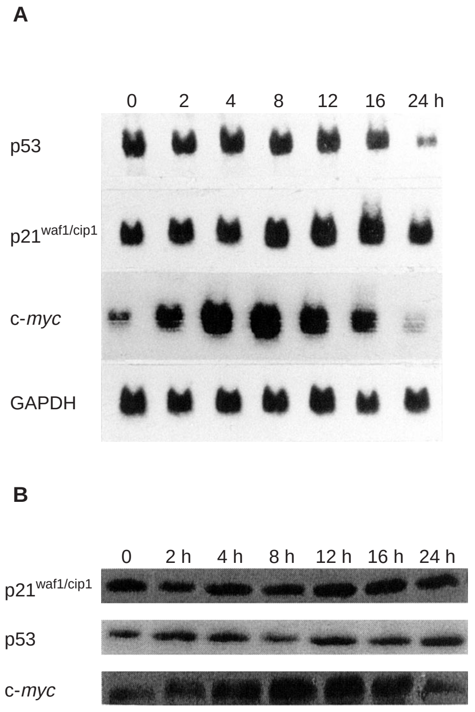

**Figure 6** The expression of p53, p21$^{waf1/cip1}$ and c-*myc* mRNA and protein in AGS cells treated by indomethacin. The AGS cells were exposed to 400 μM indomethacin for different intervals (0, 2, 4, 8, 12, 16, 24 h). The mRNA and protein level of p53, p21$^{waf1/cip1}$ and c-*myc* were determined by Northern and Western blot. The results are the representative of three different experiments. (**A**) Northern blot result. GAPDH was used as an internal control. (**B**) Immunoblot analysis

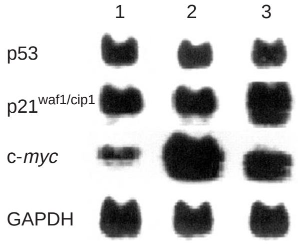

**Figure 7** The effect of TPA on the expression of p53, p21$^{waf1/cip1}$ and c-*myc* mRNA in indomethacin-induced AGS. The AGS cells were treated with indomethacin (400 μM) alone or in combination with 100 nM TPA for 8 h. The mRNA level of p53, p21$^{waf1/cip1}$ and c-*myc* were determined by Northern blot. Lane 1, control; lane 2, 400 μM indomethacin; lane 3, 400 μM indomethacin + 100 nM TPA. The house-keeping gene GAPDH was used as an internal control (the representative of three different experiments)

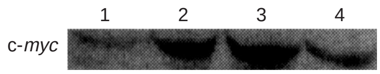

**Figure 8** Effect of oligonucleotides on c-*myc* protein expression. AGS cells were cultured with antisense or sense oligonucleotides (2 μM) for 18 h before incubation with 400 μM indomethacin for another 8 h, after which cell lysates was collected and c-*myc* protein was determined by Western blotting. Lane 1, control; lane 2, 400 μM indomethacin; lane 3, 400 μM indomethacin+2 μM sense oligos; lane 4, 400 μM indomethacin+2 μM antisense. This experiment was repeated three times and one representative result is shown in this figure cancer cells in a concentration- and time-dependent fashion. Moreover, aspirin and indomethacin induced apoptosis in the AGS cancer cells. Indomethacin showed more potent growth inhibition on AGS cells compared with aspirin. Similarly, apoptosis was induced by indomethacin at a lower concentration than aspirin. These findings are consistent with those reported in colon cancer and other cell lines (Lu et al, 1995; Shiff et al, 1996). Apoptosis is often accompanied by growth arrest. Cell cycle checkpoints may acts as a toggle switch between proliferation and death by sensing deregulation of the cell cycle (King and Cidlowski, 1995). In colon cancer cells, the apoptotic effect of NSAIDs correlated with the arrest of cells at $G_0/G_1$ phase (Hanif et al, 1996; Shiff et al, 1996). We failed to demonstrate any effect of aspirin or indomethacin on the AGS cell cycle phase distribution by using the FACS analysis. Thus, apoptosis may be the chief mechanism responsible for the inhibition of cell growth in NSAID-treated AGS cancer cells.

Although the role of NSAIDs in the induction of apoptosis of colon cancer cells are reported (Hanif et al, 1996; Shiff et al, 1996), the intracellular molecular events involved are still by far unknown. One potential molecular target for NSAIDs is cyclo-oxygenases (COXs), a key enzyme in prostaglandin biosynthesis. Interestingly, sulindac sulphone (an active metabolite of sulindac), which induced apoptosis in colon carcinoma cells HT29, is independent of inhibiting prostaglandin biosynthesis (Hanif et al, 1996). In our present study, we found that prolonged incubation with a PKC activator significantly reduced the apoptotic effect of indomethacin in the AGS cells. Thus, the protein kinase C pathway may be associated with NSAID-induced apoptosis in gastric cancer cells.

A high prevalence of p53 mutation has been detected in gastric cancer (Uchino et al, 1993; Triantafillou et al, 1996). In indomethacin-treated AGS cells, we did not observe any change in p53 mRNA and protein expression. The p53 target gene *p21*$^{waf1/cip1}$ also showed no change after the treatment with indomethacin, which might explain the unaltered cell cycle phase distribution. In HT-29 colon cancer cells, however, both sulindac and sulindac sulphide increased the level of *p21*$^{waf1/cip1}$ expression but reduced the level of mutant p53 (Goldberg et al, 1996). The differential regulation of tumour-suppressor genes in relation to apoptosis in cells from different gastrointestinal sites needs to be explored.

c-*myc* mRNA is overexpressed in most cases of primary gastric carcinoma. Metastatic lesions also have high levels of expression, which suggests that overexpression of c-*myc* may play a role in gastric carcinogenesis and tumour metastasis (Onoda et al, 1996). Our results showed that c-*myc* gene expression was up-regulated at both mRNA and protein level, preceding the onset of apoptosis after treatment with indomethacin. Down-regulation of the c-*myc* overexpression by antisense oligonucleotides inhibited the indomethacin-induced apoptosis of AGS cells, which suggested that c-*myc* was involved in indomethacin-induced apoptosis. Further evidence came from the down-regulation of c-*myc* after TPA co-treatment, which also reversed indomethacin-induced apoptosis. c-*myc*-mediated apoptosis in epithelial cells could be p53 dependent or independent (Sakamuro et al, 1995). AGS cells express wild-type p53 (Matozaki et al, 1992). After treatment with indomethacin, p53 mRNA remained unchanged, although the c-*myc* mRNA was markedly increased. It has been shown that normal levels of wild-type p53 in the fibroblast system are sufficient to confer susceptibility to c-*myc*-mediated apoptosis (Wagner

 © Cancer Research Campaign 1999

et al, 1994). Further study is needed to better define the interaction of the two genes in apoptosis.

We found that TPA could reduce the proportion of AGS cells at $G_0/G_1$ phase and increase the cells at $G_2$/M phase in the presence or absence of indomethacin. Usually, cells progress to late $G_1$ phase before apoptosis takes place. Cell cycle arrest before this stage delays or blocks apoptosis, whereas arrest after this promotes apoptosis (Meikrantz et al, 1994). It may be possible that TPA inhibits indomethacin-induced apoptosis via modulation of cell cycle kinetics. In cells treated with PKC activator in the presence of indomethacin, $p21^{waf1/cip1}$ protein expression was increased, but not accompanied by an increase in p53 protein expression. Therefore, activation/down-regulation of protein kinase C could lead to overexpression of $p21^{waf1/cip1}$ in gastric cancer cells that is independent of p53. This finding agrees with previous studies showing that $p21^{waf1/cip1}$ can be induced through p53-independent mechanisms by TPA in other cell types (Michieli et al, 1994). $p21^{waf1/cip1}$ has been commonly associated with the $G_1$ check point. However, it has been shown to accumulate cells in $G_2$/M phase in some cell types treated with TPA (Tchou et al, 1996). In our present study, overexpression of $p21^{waf1/cip1}$ may be associated with the accumulation of cells at $G_2$/M phase after TPA treatment.

We conclude from our experiments that both aspirin and indomethacin induce apoptosis in AGS gastric cancer cells, which may account for their growth inhibitory effects. The modulation of PKC activity and down-regulation of c-*myc* may block these effects. We believe that further understanding of the NSAID-induced apoptosis in gastric cancer can lead to some insight into the carcinogenesis and chemoprevention of the second most common cancer in the world.

### ACKNOWLEDGEMENTS

This work was supported in part by CRCG research grant 337/041/0080. We are grateful to Mr KH Ko at the Department of Pathology, the University of Hong Kong, for his kind assistance in FACS scan and the data analysis.

### REFERENCES

Askew DS, Ashmun RA, Simmons BC and Cleveland JL (1991) Constitutive c-*myc* expression in an IL-3-dependent myeloid cell line suppresses cell cycle arrest and accelerates apoptosis. *Oncogene* **6**: 1915–1922

Ausubel FM, Brent R, Kingston RE, Moore DD, Seidman JG, Smith JA and Struhl K (1995) *Current Protocols in Molecular Biology*, Vol. 1 4.9.1. John Wiley & Sons: USA

Carmichael J, Mitchell JB, DeGraff WG, Gamson J, Gazdar AF, Johnson BE, Glatstein E and Minna JD (1988) Chemosensitivity testing of human lung cancer cell lines using the MTT assay. *Br J Cancer* **57**: 540–547

Clemens MJ, Trayner I and Menaya J (1992) The role of protein kinase C isoenzymes in the regulation of cell proliferation and differentiation. *J Cell Sci* **103**: 881–887

Darzynkiewicz Z, Bruno S, Del Bino S, Gorczyca W, Hotz MA, Lassota P and Traganos F (1992) Features of apoptotic cells measured by flow cytometry. *Cytometry* **13**: 795–808

Deacon EM, Pongracz J, Griffiths G and Lord JM (1997) Isoenzymes of protein kinase C: differential involvement in apoptosis and pathogenesis. *Mol Pathol* **50**: 124–131

El-Deiry WS, Harper JW, O'Connor PM, Velculescu VE, Canman CE, Jackman J, Pietenpol JA, Burrell M, Hill DE, Wang Y, Wiman KG, Mercer WE, Kastan MB, Kohn KW, Elledge SJ, Kinzler KW and Vogelstein B (1994) WAF1/CIP1 is induced in p53-mediated G1 arrest and apoptosis. *Cancer Res* **54**: 1169–1174

Elder DJ, Hague A, Hicks DJ and Paraskeva C (1996) Differential growth inhibition by the aspirin metabolite salicylate in human colorectal tumor cell lines: enhanced apoptosis in carcinoma and in vitro-transformed adenoma relative to adenoma cell lines. *Cancer Res* **56**: 2273–2276

Evans GI, Wyllie AH, Gilbert CS, Littlewood TD, Land H, Brooks M, Waters CM, Penn LZ and Hancock DC (1992) Induction of apoptosis in fibroblasts by c-myc protein. *Cell* **69**: 119–128

Giardiello FM, Hamilton SR, Krush AJ, Piantadosi S, Hylind LM, Celano P, Brooker SV, Robinson CR and Offerhaus GJ (1993) Treatment of colonic and rectal adenomas with sulindac in familial adenomatous polyposis. *N Engl J Med* **328**: 1313–1316

Giovannucci E, Rimm EB, Stampfer MJ, Colditz GA, Ascherio A and Willett WC (1994) Aspirin use and the risk for colorectal cancer and adenoma in male health professionals. *Ann Intern Med* **121**: 241–246

Goldberg Y, Nassif II, Pittas A, Tsai LL, Dynlacht BD, Rigas B and Shiff SJ (1996) The anti-proliferative effect of sulindac and sulindac sulfide on HT-29 colon cancer cells: alterations in tumor suppressor and cell cycle-regulatory proteins. *Oncogene* **12**: 893–901

Grant S, Jarvis WD, Swerdlow PS, Turner AJ, Traylor RS, Wallace HJ, Lin PS, Pettit GR and Gewirtz DA (1992) Potentiation of the activity of 1-β-D-arabinofuranosylcytosine by the protein kinase C activator bryostatin 1 in HL-60 cells: association with enhanced fragmentation of mature DNA. *Cancer Res* **52**: 6270–6278

Gridley G, McLaughlin JK, Ekbom A, Klareskog L, Adami HO, Hacker DG, Hoover R and Fraumeni Jr JF (1993) Incidence of cancer among patients with rheumatoid arthritis. *J Natl Cancer Inst* **85**: 307–311

Hale AJ, Smith CA, Sutherland LC, Stoneman VE, Longthorne VL, Culhane AC and Williams GT (1996) Apoptosis: molecular regulation of cell death. *Eur J Biochem* **236**: 1–26

Hall PA, Coates PJ, Ansari B and Hopwoon D (1994) Regulation of cell number in the mammalian gastrointestinal tract: the importance of apoptosis. *J Cell Sci* **107**: 3569–3577

Hanif R, Pittas A, Feng Y, Koutsos MI, Qiao L, Staiano-Coico L, Shiff SJ and Rigas B (1996) Effects of nonsteroidal anti-inflammatory drugs on proliferation and on induction of apoptosis in colon cancer cells by a prostaglandin-independent pathway. *Biochem Pharmacol* **52**: 237–245

Isomaki HA, Hakulinen T and Joutsenlahti U (1978) Excess risk of lymphomas, leukemia and myeloma in patients with rheumatoid arthritis. *J Chronic Dis* **31**: 691–696

Jarvis WD, Tuner AJ, Povirk LF, Traylor RS and Grant S (1994) Induction of apoptotic DNA fragmentation and cell death in HL-60 human promyelocytic leukemia cells by pharmacological inhibitors of protein kinase C. *Cancer Res* **54**: 1707–1714

King KL and Cidlowski JA (1995) Cell cycle and apoptosis: common pathways to life and death. *J Cell Biochem* **58**: 175–180

Li Y, Davis KL and Sytkowski AJ (1996) Protein kinase C-ε is necessary for erythropoietin's up-regulation of c-*myc* and for factors-dependent DNA synthesis. Evidence for discrete signals for growth and differentiation. *J Biol Chem* **271**: 27025–27030

Liebermann DA, Hoffman B and Steinman RA (1995) Molecular controls of growth arrest and apoptosis: p53-dependent and independent pathways. *Oncogene* **11**: 199–210

Lotem J and Sachs L (1993) Regulation by bcl-2, c-myc, and p53 of susceptibility to induction of apoptosis by heat shock and cancer chemotherapy compounds in differentiation-competent and -defective myeloid leukemia cells. *Cell Growth Differ* **4**: 41–47

Lu XJ, Xie WL, Reed D, Bradshaw WS and Simmons DL (1995) Nonsteroidal antiinflammatory drugs cause apoptosis and induce cyclooxygenases in chicken embryo fibroblast. *Proc Natl Acad Sci USA* **92**: 7961–7965

Lucas M and Sanchez-Margalet V (1995) Protein kinase C involvement in apoptosis. *Gen Pharmacol* **26**: 881–887

Marnett LJ (1992) Aspirin and the potential role of prostaglandins in colon cancer. *Cancer Res* **52**: 5575–5589

Matozaki T, Sakamoto C, Matsuda K, Suzuki T, Konda Y, Nakano O, Wada K, Uchida T, Nishisaki H, Nagao M and Kasuga M (1992) Missense mutation and a deletion of the p53 gene in human gastric cancer. *Biochem Biophys Res Commun* **182**: 215–223

Meikrantz W, Gisselbrecht S, Tam SW and Schlegel R (1994) Activation of cyclin A-dependent protein kinases during apoptosis. *Proc Natl Acad Sci USA* **91**: 3754–3758

Michieli P, Chedid M, Lin D, Pierce JH, Mercer WE and Givol D (1994) Induction of WAF1/CIP1 by a p53-independent pathway. *Cancer Res* **54**: 3391–3395

Onoda N, Maeda K, Chung YS, Yano Y, Matsui-Yuasa I, Otani S and Sowa M (1996) Overexpression of c-myc messenger RNA in primary and metastatic lesions of carcinoma of the stomach. *J Am Coll Surg* **182**: 55–59

© Cancer Research Campaign 1999

Rhoads RE (1975) Ovalbumin messenger ribonucleic acid. Purification and fractionation on the basis of polyadenylate content by thermal elution from oligodeoxythymidylate-cellulose. *J Biol Chem* **250**: 8088–8097

Sakamuro D, Eviner V, Elliott KJ, Showe L, White E and Prendergast GC (1995) c-*Myc* induces apoptosis in epithelial cells by both p53-dependent and p53-independent mechanisms. *Oncogene* **11**: 2411–2418

Shiff SJ, Koutsos MI, Qiao L and Rigas B (1996) Nonsteroidal antiinflammatory drugs inhibit the proliferation of colon adenocarcinoma cell: effects on cell cycle and apoptosis. *Exp Cell Res* **222**: 179–188

Smith PK, Krohn RI, Hermanson GT, Mallia AK, Gartner FH, Provenzano MD, Fujimoto EK, Goeke NM, Olson BJ and Klenk DC (1985) Measurement of protein using bicinchoninic acid. *Anal Biochem* **150**: 76–85

Spyridon L, Akira N, Hiromasa K, Katsutoshi G, Takao M, Yoshiki O, Hideo S and Hisayuki F (1994) The development of the endothelin-1-induced gastric ulcer: time sequence analysis of morphological changes. *Int J Exp Pathol* **75**: 345–355

Tchou WW, Rom WN and Tchou-Wong KM (1996) Novel form of $p21^{WAF1/CIP1/SDI1}$ protein in phorbol ester-induced $G_2$/M arrest. *J Biol Chem* **271**: 29556–29560

Thun MJ, Namboodiri MM, Calle EE, Flanders WD and Heath Jr CW (1993) Aspirin use and risk of fatal cancer. *Cancer Res* **53**: 1322–1327

Triantafillou NG, Grosman IM and Verma RS (1996) Genomania of p53 protein in gastric cancer. *J Clin Gastroenterol* **22**: 170–173

Uchino S, Noguchi M and Ochiai A (1993) p53 mutation in gastric cancer: a genetic model for carcinogenesis is common to gastric and colorectal cancer. *Int J Cancer* **54**: 759–764

Wagner AJ, Kokontis JM and Hay N (1994) Myc-mediated apoptosis requires wild-type p53 in a manner independent of cell cycle arrest and the ability of p53 to induce $p21^{waf1/cip1}$. *Genes Dev* **8**: 2817–2830

Xiong Y, Hannon GJ, Zhang H, Casso D, Kobayashi R and Beach D (1993) p21 is a universal inhibitor of cyclin kinases. *Nature* **366**: 701–704

 *© Cancer Research Campaign 1999*
